# Molecular Imaging Reveals a Progressive Pulmonary Inflammation in Lower Airways in Ferrets Infected with 2009 H1N1 Pandemic Influenza Virus

**DOI:** 10.1371/journal.pone.0040094

**Published:** 2012-07-20

**Authors:** Colleen B. Jonsson, Jeremy V. Camp, Albert Wu, Huaiyu Zheng, Jennifer L. Kraenzle, Ashley E. Biller, Carol D. Vanover, Yong-Kyu Chu, Chin K. Ng, Mary Proctor, Leslie Sherwood, Marlene C. Steffen, Daniel J. Mollura

**Affiliations:** 1 Center for Predictive Medicine for Biodefense and Emerging Infectious Diseases, University of Louisville, Louisville, Kentucky, United States of America; 2 Department of Microbiology and Immunology, University of Louisville, Louisville, Kentucky, United States of America; 3 Center for Infectious Disease Imaging, Department of Radiology and Imaging Sciences, National Institutes of Health, Bethesda, Maryland, United States of America; 4 Department of Radiology, University of Louisville, Louisville, Kentucky, United States of America; 5 Department of Research Resources Facilities, University of Louisville, Louisville, Kentucky, United States of America; Lovelace Respiratory Research Institute, United States of America

## Abstract

Molecular imaging has gained attention as a possible approach for the study of the progression of inflammation and disease dynamics. Herein we used [^18^F]-2-deoxy-2-fluoro-D-glucose ([^18^F]-FDG) as a radiotracer for PET imaging coupled with CT (FDG-PET/CT) to gain insight into the spatiotemporal progression of the inflammatory response of ferrets infected with a clinical isolate of a pandemic influenza virus, H1N1 (H1N1pdm). The thoracic regions of mock- and H1N1pdm-infected ferrets were imaged prior to infection and at 1, 2, 3 and 6 days post-infection (DPI). On 1 DPI, FDG-PET/CT imaging revealed areas of consolidation in the right caudal lobe which corresponded with elevated [^18^F]-FDG uptake (maximum standardized uptake values (SUVMax), 4.7–7.0). By days 2 and 3, consolidation (CT) and inflammation ([^18^F]-FDG) appeared in the left caudal lobe. By 6 DPI, CT images showed extensive areas of patchy ground-glass opacities (GGO) and consolidations with the largest lesions having high SUVMax (6.0–7.6). Viral shedding and replication were detected in most nasal, throat and rectal swabs and nasal turbinates and lungs on 1, 2 and 3 DPI, but not on day 7, respectively. In conclusion, molecular imaging of infected ferrets revealed a progressive consolidation on CT with corresponding [^18^F]-FDG uptake. Strong positive correlations were measured between SUVMax and bronchiolitis-related pathologic scoring (Spearman’s ρ = 0.75). Importantly, the extensive areas of patchy GGO and consolidation seen on CT in the ferret model at 6 DPI are similar to that reported for human H1N1pdm infections. In summary, these first molecular imaging studies of lower respiratory infection with H1N1pdm show that FDG-PET can give insight into the spatiotemporal progression of the inflammation in real-time.

## Introduction

In March of 2009, an outbreak of a novel variant of H1N1 influenza A virus was reported in cases of influenza illness in Mexico [1]. By June 11, the World Health Organization raised the pandemic alert level to its highest level, declaring the first influenza pandemic in over 40 years [1]. Unlike seasonal influenza viruses, this novel H1N1 pandemic strain (H1N1pdm) tended to affect younger healthier populations and had an increased risk of morbidity and mortality [2]–[4] with 12–30% of the population developing clinical influenza, 4% of those requiring hospital admission, and 1 in 5 requiring critical care [5]. In general, however, infection of the H1N1pdm was relatively mild in most persons, although a fatal viral pneumonia with acute respiratory distress syndrome occurred in approximately 18,000 cases.

In contrast to seasonal influenza in human cases, H1N1pdm infections showed a tropism for the lung similar to H5N1 [6]. The ability of H1N1pdm viruses to infect the lower respiratory track has been attributed to a broader specificity in the binding of the viral hemagglutinin (HA) to α2-3- in addition to α2-6-linked sialic acid (SA) receptors [7], [8]. It is reasonable that the lung tropism of the H1N1pdm contributed to the severity of disease in those individuals with preexisting complications such as asthma and chronic obstructive pulmonary disease (COPD) [6], [9]–[12]. Data from limited human autopsies and animal studies of various pandemic strains also suggest contribution of the host innate immune response and the virus in the progression of disease [13]–[16].

Molecular imaging can potentially play a strong role in basic infectious disease research and clinical response by providing a noninvasive, spatiotemporal measurement of viral infection and host inflammation [17], [18]. To explore the potential utility of molecular imaging in influenza infection, we chose the ferret (*Mustela putorius furo*) model. Ferrets have been used as an animal model of influenza infection and pathogenesis since 1934, when they were reported to develop an acute respiratory tract illness when exposed to influenza viruses from humans and swine [19]. In contrast to mice, the ferret can be infected by human isolates without adaptation and display signs and symptoms of infection such as sneezing and nasal secretions that are similar to what is seen in humans [20]–[23]. The ferret is an attractive model for imaging influenza pulmonary infections given the ferret’s long trachea, large lung capacity, and bronchiolar branching. These anatomical features can potentially bridge imaging with histopathologic evaluation. Finally, the ferret more closely mimics humans in distribution of sialic acid (SA) receptors in the respiratory tract with higher α-2-6 SA in the upper respiratory tract and SA with α-2-3- in the lower [24].

Historically, plain film (x-ray) radiography and computed tomography (CT) have been useful for clinical assessments of influenza disease severity in clinical cases [25], [26]. These imaging modalities are limited by characterizing only anatomic changes in the lung parenchyma, such as ground-glass opacity (GGO) and consolidations, which represent different degrees of interstitial and alveolar filling by cells, edema, and inflammatory exudate [27]. In contrast, positron emission tomography (PET) imaging with the radiotracer, [^18^F]-2-fluoro-2-deoxy-D-glucose ([^18^F]-FDG), can provide data on metabolic activity of cells by measuring sites of increased glycolysis from leukocyte chemotaxis and accumulation, and provide increased sensitivity in detection of cells during inflammation. [^18^F]-FDG, an analog of glucose that is moved into cells via facilitated transport, is commonly used in PET imaging as a radiotracer in clinical and basic science research. Recent studies have demonstrated the utility of the [^18^F]-FDG in assessment of infectious disease burden in animal models of schistosomiasis and tuberculosis [28]–[30]. PET/CT has been used in the assessment of one hospitalized H1N1pdm patient and revealed an intense inflammatory response [31]. The uptake of [^18^F]-FDG in humans and animals suggests the predominant presence of activated neutrophils [32]–[35]. In studies of mice infected with influenza A virus, neutrophils play a critical role in protection and recovery from infection, and participate in the process of adaptive immunity to the virus [36]. Coupled with molecular virology and pathology, molecular imaging with important probes of disease has enormous potential to reveal early critical factors that contribute to the clinical progression of illness as well as accelerate screening, the efficacy and mechanistic studies of vaccines and antiviral therapies [17], [18].

To test the hypothesis that FDG-PET/CT imaging could reveal the spatiotemporal nature of H1N1pdm inflammation and disease progression, we chose the ferret model of H1N1pdm influenza infection. Further, for these studies we chose a low passage clinical isolate, A/Kentucky/180/2010 or KY/180, herein, which has a change in the HA1 gene, D222G, which correlates with increased severity of disease in patient cases from several countries [37]–[40]. In mice, infection with H1N1pdm engineered to include this change show increased viral titers and pathology, however, in ferrets there do not seem to be any major differences in clinical signs, transmissibility or pathogenicity [41], [42]. Our results show for the first time, the spatiotemporal progression of inflammation with CT and PET using [^18^F]-FDG in ferrets infected with H1N1pdm in conjunction with histopathology and viral titers over a seven day period. Importantly, the extensive areas of patchy GGO and consolidation seen in the ferret model at 6 days post-infection (DPI) are similar to that reported for human H1N1pdm infections [31]. In vivo imaging with these modalities for anatomic (CT) and molecular (PET) data suggests increased pulmonary inflammation as the amount of circulating virus becomes undetectable. These results suggest that molecular imaging will be a great asset in gaining insight into the temporal and spatial progression of the inflammatory process caused by influenza virus infection.

## Results

### Characterization of KY/180 in Female Ferrets

Due to the size of the Siemens Trimodal gantry for PET/CT imaging we chose four month old females rather than male ferrets. The pandemic H1N1 isolate KY/180 employed in these studies was isolated from nasal swab sample provided by the Severe Influenza Pneumonia Surveillance project, a clinical study of hospitalized patients with influenza pneumonia in Kentucky. The patient had a severe course of influenza disease and died after 19 days. Sequencing of the HA1 gene from this isolate revealed the D222G mutation, which has been associated with severe disease in human cases [37]–[40]. The second passage of the KY/180 seed was employed in the characterization of infection in the female ferrets. The 50% infectious dose in female ferrets was determined to be 10^0.07^ TCID_50_ (data not shown). In group 1, six ferrets were mock infected with PBS. In group 2, six ferrets were infected intranasally (i.n.) with KY/180 with a 0.5 ml dose of 0.5×10^5.7^ TCID_50_ per naris. Ferrets were monitored for temperature for 10 DPI, and for body weight and clinical symptoms for 28 DPI. Two animals in each group were euthanized on days 2, 14 and 18 to determine virus and HI titers in blood, lung and several additional organs at 2 DPI.

**Figure 1:**
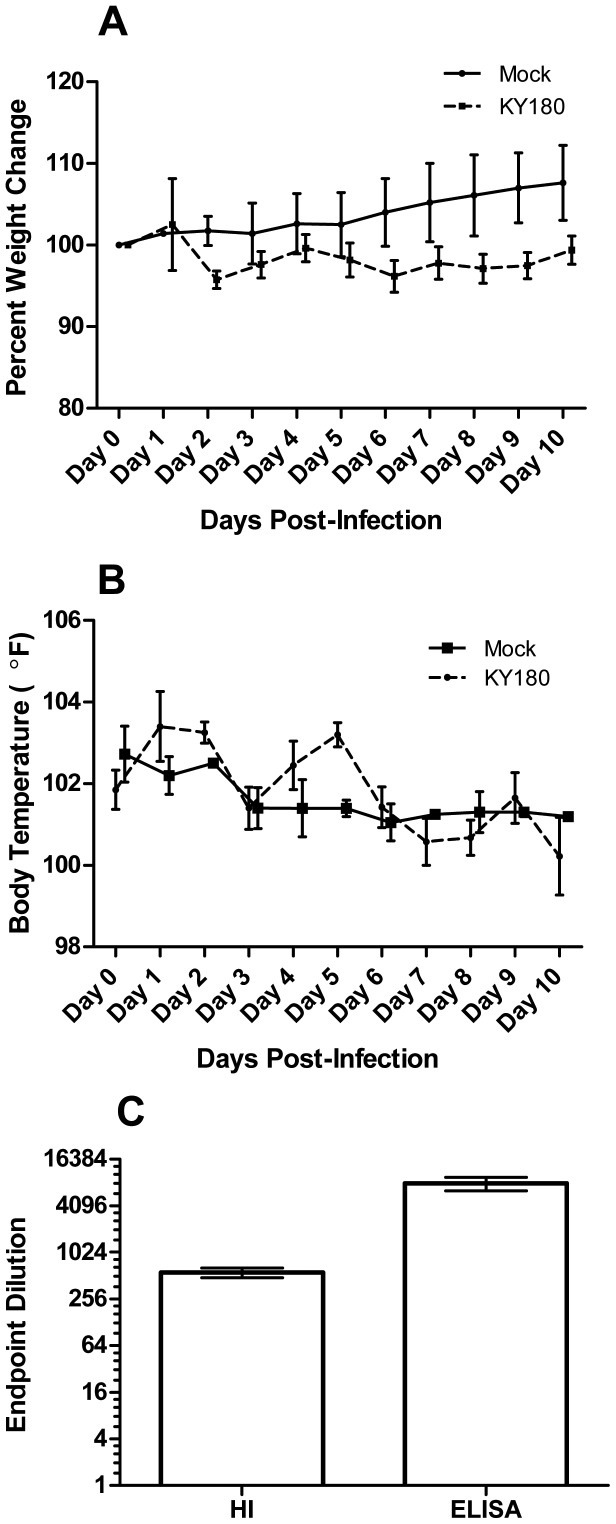
Characterization of KY/180 in female ferrets. A cohort of six control and six KY/180-infected ferrets were examined for A) body weight and B) temperature over a period of 10 days. C) Blood was examined for the presence of HI and antibody titers at 14 days.

In figure 1A, the body weight changes are shown for mock- and KY/180-infected ferrets. Body weight showed a drop on day 2 where the weight remained for the remainder of the study. Figure 1B shows the average temperatures of the mock- and KY/180-infected ferrets for the first 10 DPI. Temperature peaked on days 1 and 5 for KY/180 infected animals with a mean temperature of 103.4°F (SD = 1.71°F) and 103.2°F (SD = 0.52°F), respectively. The average hemagglutinin inhibition (HI) serum antibody titer in the blood on day 14 was 540 (reciprocal dilution) and the average total serum influenza-specific IgG by ELISA was 7610 (reciprocal dilution) (Fig. 1C). In studies to determine the infectious dose, additional tissues were taken from animals infected at with KY/180 with a 0.5 ml dose of 0.5×10^4.7^ TCID_50_ per naris. On day 2, viral titers were the highest in the nasal turbinates (10^6.25^ TCID_50_/g) followed by the caudal lung (10^6.0^ TCID_50_/g) and trachea (10^3.75^ TCID_50_/g). The lowest levels of virus were observed in the cranial lobe of the lung (10^3.2^ TCID_50_/g). Viral titers were measured by TCID_50_ in nasal turbinates, trachea, right cranial lobe of the lung, right caudal lobe, brain, liver, spleen, kidney, duodenum, jejunoilieum, colon and rectum. KY/180 was detected in jejunum in one animal (10^2.0^ TCID_50_/g), but was not detected in any other tissues (data not shown).

### FDG-PET/CT Imaging of the H1N1pdm-infected Ferrets Show Progressive Inflammation

Female Fitch ferrets were divided into five groups, with two per group of animals that were mock-infected with PBS (group 1) or intranasally infected with KY/180 (groups 2–5), in a 0.5 ml dose of 0.5×10^6.0^ TCID_50_ per naris, on day 0 (Table 1). Group 5 was the only group that was imaged each day; while groups 2–4 were imaged and sacrificed on days 1, 2 and 3 post-infection. This study design permitted evaluation of the progression of imaging with infection and pathology in two animals each day as well as continuous imaging of the lungs in one cohort over the seven-day time-period.

**Table 1:** Study design for ferret imaging and sample collection*.

Group	AnimalID	Day 0	Day 1	Day 2	Day 3	Day 6	Day 7
1	2206/7	£	†	†			
	2210/3				†		†
2	2208		£,†				
	2209		£,†				
3	2202			£,†			
	2204			£,†			
4	2211				£,†		
	2212				£,†		
5	2213	£	£	£	£	£	†
	2214	£	£	£	£	£	†

† Date for necropsy.

£ Date for imaging.

* Swab samples were collected for viral shedding daily and upon necropsy.

**Figure 2:**
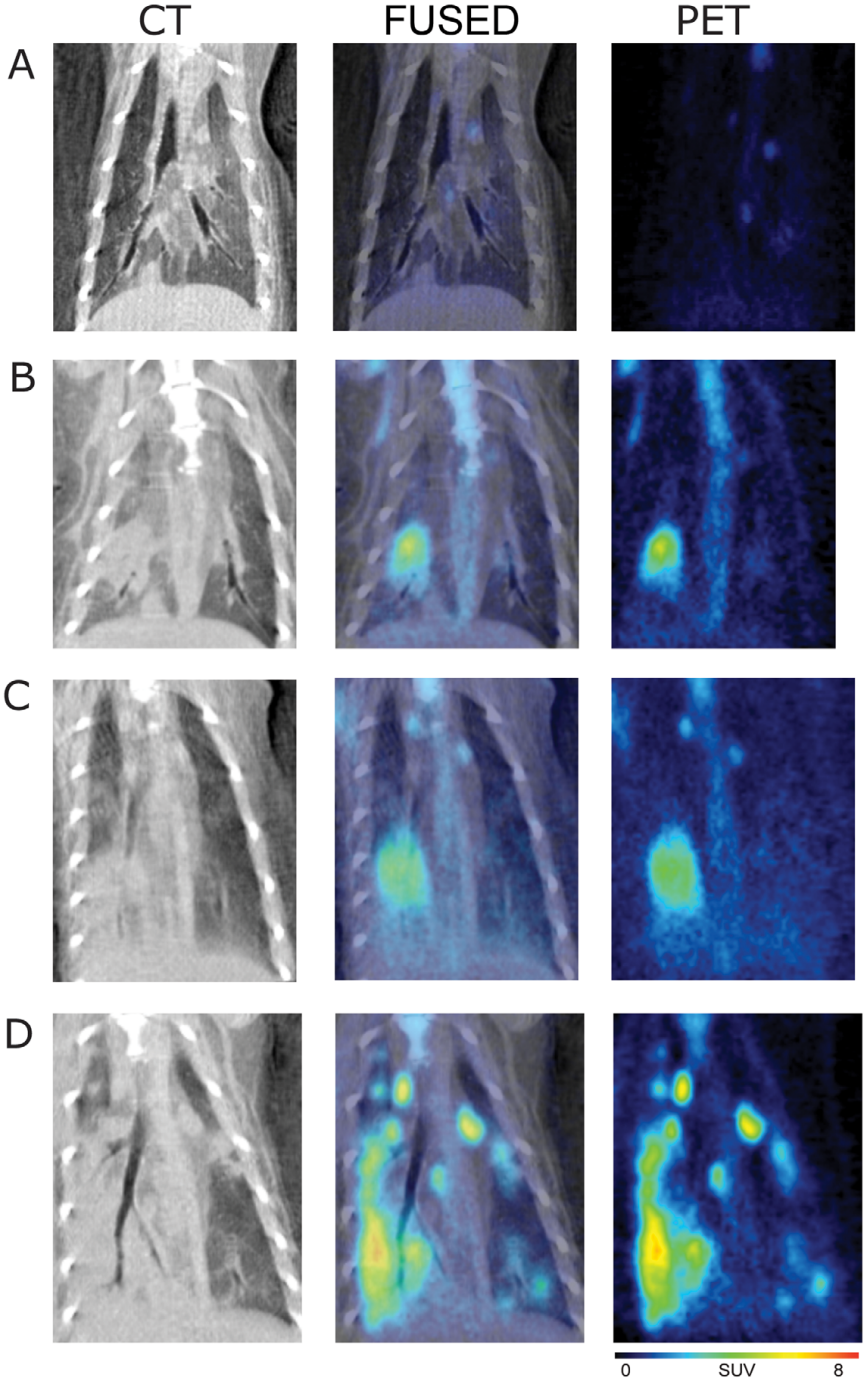
18F-FDG PET, CT, and PET/CT fusion images of the thorax in H1N1pdm-infected ferret 2213. A) Day 0 shows background activity in the lung with minimal uptake in the mediastinal and subcarinal lymph nodes. B) Day 1 post-infection demonstrates an area of developing consolidation in the right caudal lobe corresponding to increased radiotracer uptake. C) By day 3, the consolidation appears in the right caudal lobe and into the left caudal lobe. D) By day 6, there are multiple lesions in the lung parenchyma bilaterally with intense radiotracer uptake.

**Figure 3:**
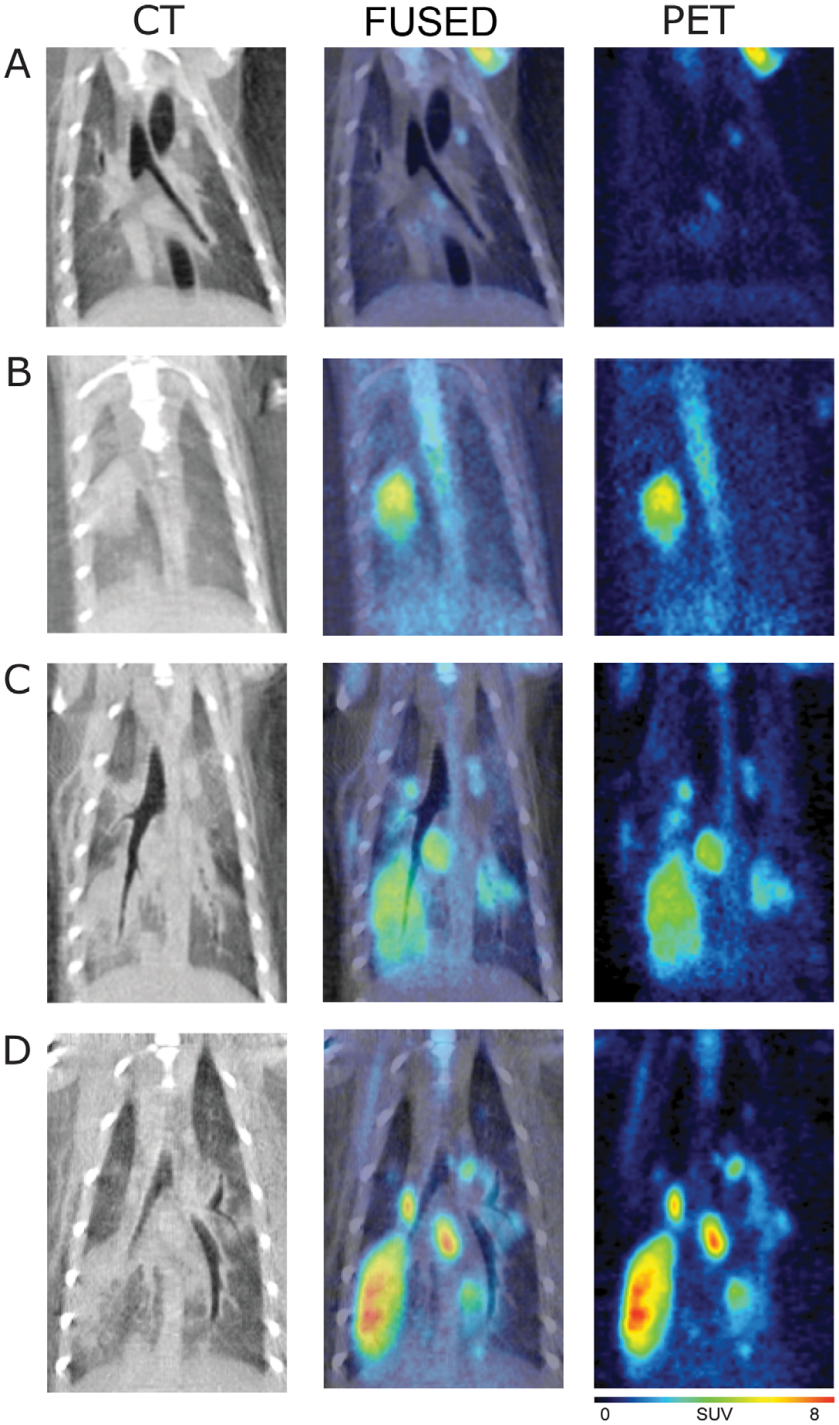
18F-FDG PET, CT, and PET/CT fusion images of the thorax in H1N1pdm-infected ferret 2214. A) Day 0 shows background activity in the lung with minimal uptake in the mediastinal and subcarinal lymph nodes. B) Day 1 post-infection demonstrates an area of developing consolidation in the right caudal lobe corresponding to increased radiotracer uptake. C) By day 3, the consolidation has spread in the right caudal lobe and into the left caudal lobe. D) By day 6, there are multiple lesions in the lung parenchyma bilaterally with intense radiotracer uptake.

FDG-PET and CT images of the H1N1pdm-infected and mock-infected ferrets were successfully obtained and fused for two ferrets on days 1, 2, 3 and 6 (Fig. 2 and 3). Volumes of interest (VOI) and corresponding maximum standardized uptake values (SUVMax) were generated for any metabolically active lesions in the lung as well as background activity in the lungs, liver, heart, thymus, and thoracic lymph nodes. Baseline imaging prior to infection showed no focal areas of lung consolidation on CT and background standard uptake values (SUVMax) of the [^18^F]-FDG levels ranged from 0.7–1.0 for PET (Fig. 2A and Fig. 3A). Each figure shows the one two-dimensional coronal plane that were standardized across days to provide a similar orientation and do not necessarily represent the SUVMax as that plane may be out of view.

By 1 DPI, an area of consolidation was identified on CT in the right caudal lobe with corresponding radiotracer uptake on PET (Fig. 2B, SUVMax of 4.7). Consolidative areas in the right caudal lobe increased by day 2, with a persistently elevated SUVMax of 3.1(data not shown). By day 3, the consolidation increased in the right caudal lobe (Fig. 2C and Fig. 3C, SUVMax of 3.7 and 4.4, respectively) and also appeared in the left caudal lobe (SUVMax of 3.2) of ferret 2214 (Fig. 3C). By 6 DPI, there were widespread areas of patchy consolidation on CT with multiple areas of increased radiotracer uptake in both ferrets in caudal and cranial lobes (Fig. 2D and Fig. 3D, SUVMax of 6.0 and 7.6 on the right, 4.2 and 4.6 on the left, respectively). These results suggest that inflammation progresses into the lower respiratory airways after infection into the upper part of the lower respiratory system. A ferret from the uninfected cohort was also imaged on day 6, with no focal appearance of consolidation on CT and no evidence of increased [^18^F]-FDG uptake on PET (image not shown, background SUV of 0.6).

### Viral shedding and Replication of the Nasal Turbinates and Lung Tissues

To measure viral shedding, each day each ferret was swabbed in the nasal, throat and fecal passages and the viral titer was measured by TCID_50_ (Table 2). The highest levels of viral shedding were measured in the throat swabs. Nasal swabs also showed viral shedding for most animals, while the presence of virus in rectal swabs was low although detectable in a few animals.

**Table 2:** Viral Shedding*.

Group	Animal ID	Area	Day 1	Day 2	Day 3	Day 7
1	2206	Nasal	0			
		Throat	0			
		Rectal	0			
	2207	Nasal		0		
		Throat		0		
		Rectal		0		
	2210	Nasal			0	
		Throat			0	
		Rectal			0	
	2203	Nasal				0
		Throat				0
		Rectal				0
2	2208	Nasal	0			
		Throat	4.7			
		Rectal	2.2			
	2209	Nasal	4.7			
		Throat	5.3			
		Rectal	0			
3	2202	Nasal	3.0	3.5		
		Throat	3.5	3.5		
		Rectal	1.2	0		
	2204	Nasal	0	3.0		
		Throat	3.5	3.5		
		Fecal	0	0		
4	2211	Nasal	0	0	0	
		Throat	3.8	4.7	4.3	
		Rectal	1.0	0	0	
	2212	Nasal	0	3.5	0	
		Throat	4.5	4.3	3.5	
		Rectal	1.0	0	0	
5	2213	Nasal	3.8	4.3	3.5	1.5
		Throat	5.5	2.7	5.0	2.0
		Rectal	0	0	0	0
	2214	Nasal	4.0	3.7	3.3	0
		Throat	6.7	3.7	5.3	0
		Rectal	2.7	0	2.3	0

* Numerical values are log base 10.

**Figure 4:**
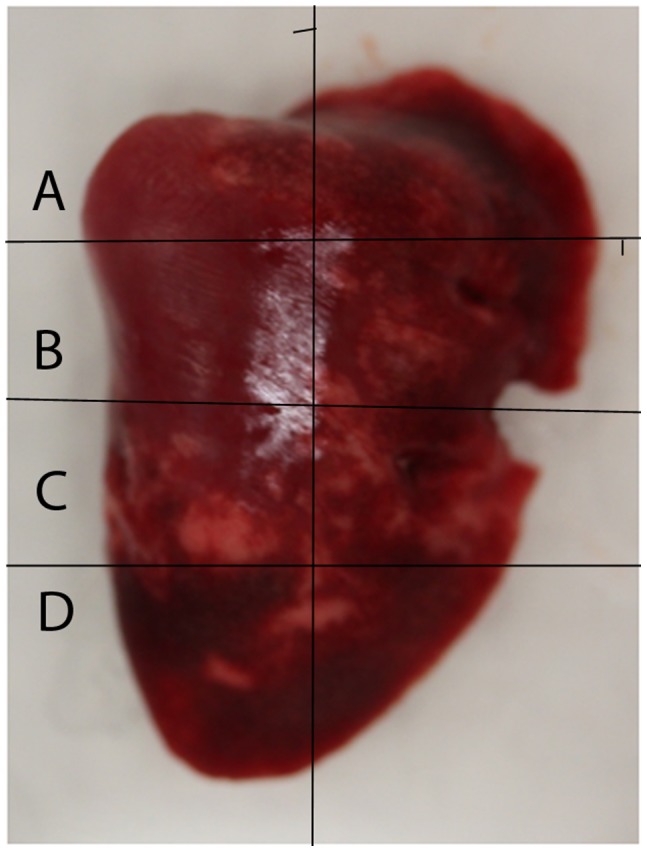
Right caudal bronchus of ferret. The right caudal lobe of each ferret was divided into 4 sections, A (top), B (middle), C (middle-lower) and D (bottom) for measurement of viral replication. Please refer to table 3 for viral replication titers measured from each tissue section.

Replication of H1N1pdm in nasal turbinates and lungs were determined post-mortem from the right caudal lobe of the lung taken on euthanasia (Table 3). Four sections were taken per lobe to provide greater insight into the spread of the virus in the tissue (Fig. 4). High levels of virus were detected in all nasal turbinate samples at 1, 2, and 3 DPI (95% C.I. = 5.43+/−1.00 TCID_50_/mL). Virus was also detected in a majority of lung sections from 1, 2, and 3 DPI. It was absent in the lung sections from one animal on 2 DPI, although it was present in the ferret’s nasal turbinates, suggesting that the timing of infection in this animal was slower than the others. Of note, this same animal had a focus of consolidation on CT and radiotracer uptake on PET. This observation also suggests that, while the virus may have been undetectable by TCID_50_, low levels of virus had entered the lower respiratory system. No animals on day 7 post-infection had detectable virus in the nasal turbinates or the sampled lung tissue. Virus was not detected in nasal, throat and fecal passages or the sampled lung tissue from ferrets in any of the controls.

**Table 3:** Distribution of H1N1pdm in right caudal and nasal turbinates*.

Day	Animal ID	RC A	RC B	RC C	RC D	NT
1	2208	6.7	6.5	5.0	5.7	5.7
	2209	0	6.5	0	1	6.8
2	2202	0	4.4	3.3	4.5	5.0
	2204	0	0	0	0	4.5
3	2211	4.2	0	0	2.8	6.0
	2212	5.8	6.5	3.5	6.3	4.7
7	2213	0	0	0	0	0
	2214	0	0	0	0	0

* Numerical values represent log base 10.

Abbreviation: right caudal or RC, for location of sections A through D illustrated in figure 4, pone.0040094.g007.tifnasal turbinate or NT.

### Histopathology of H1N1pdm-infected and Mock-infected Ferrets

**Figure 5:**
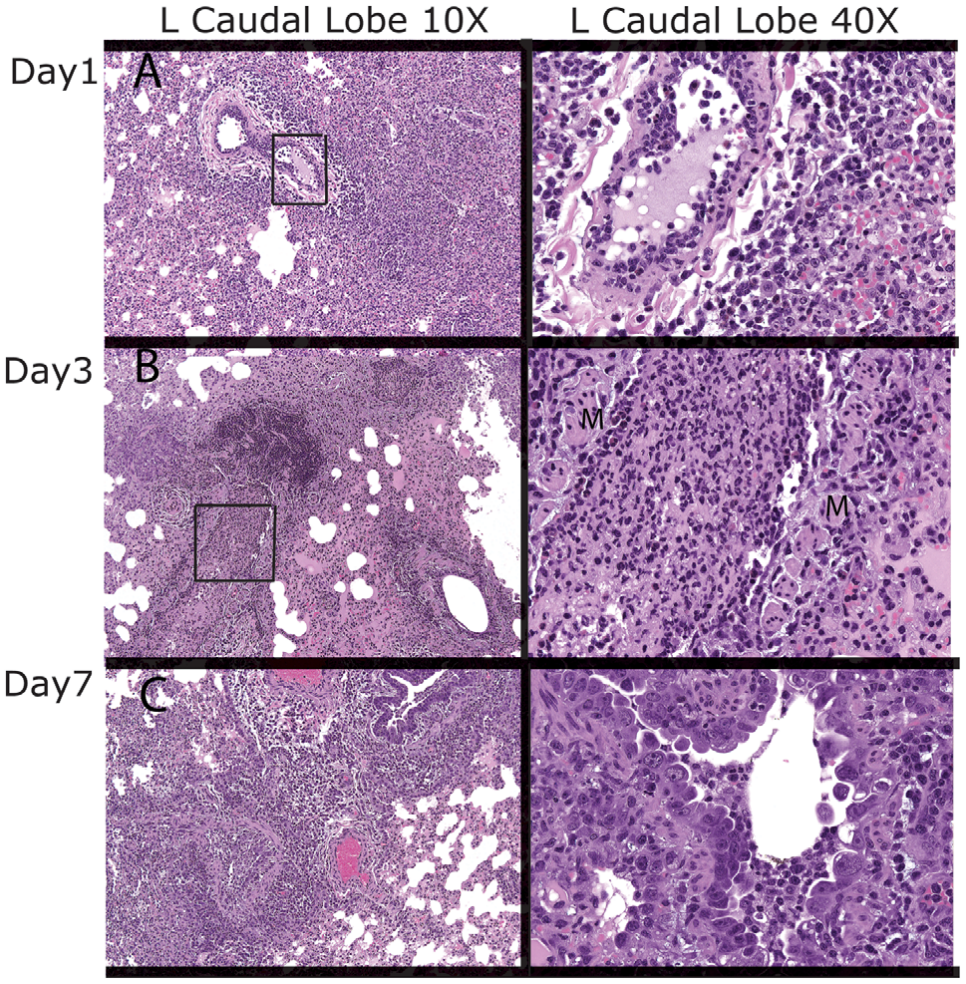
Histopathologic evaluation of the lung caudal lobe in H1N1 infected ferrets. A) On day 1, there are small foci of inflammation (10×) in animal 2208. A higher 40×magnification of the bronchiole (shown in the 10×magnification with a box) exhibits neutrophils within the lumen with loss of part of the epithelial layer. Mixed cell alveolitis surrounds the bronchiole. B) On day 3, a bronchiole with necrosupprative exudate is highlighted at 10× for animal 2212. In the 40×magnification (from the region in the 10×magnification with a box), muscle (M) defines the bronchiole from which the epithelium has been sloughed and the lumen is filled with neutrophils (necrosuppurative bronchiolitis). C) On day 7, a bronchiole with necrosuppurative exudate is surrounded by an extensive area of mixed cell alveolitis. Cytokaryomegaly is depicted by the variable large size and shape of the epithelial cells with piling up of the bronchiolar epithelium (hyperplasia) as shown in 10×image, and also visible in this bronchiole from another section of the left caudal lung lobe from animal 2214 (40× image).

Upon necropsy, all but the right caudal lobe of the ferret lung was fixed with paraformaldehyde. Following fixation, sections were taken for histopathology from the right and left cranial lobes, left caudal lobe and the middle accessory lobe. Representative photographs from slides of the left caudal lobe are shown in figure 5. The ferrets in the control group had intact bronchiolar walls with very minimal infiltration by neutrophils with the exception of the left caudal lobe from control animal 2206 sacrificed on Day 1. Possible causes of this pattern of change may be an underlying systemic vasculopathy which is typically confirmed by evaluation of other organs that were not collected (e.g., kidney, spleen, liver).

In general pulmonary lesions associated with influenza infection were roughly comparable at Days 1 and 2 and consisted of variable suppurative or necrosuppurative bronchiolitis and mixed cell alveolitis at minimal to moderate severity levels. By 1 DPI, there were some small foci of inflammation without much infiltration of the bronchi or bronchioles. There was an increased severity of inflammatory findings in lung lobes from infected ferrets on day 3. Specifically, more extensive infiltration of neutrophils can be seen within the bronchiolar lumen, along with necrosupprative bronchiolitis and mixed cell alveolitis. At Day 7, lesions observed in the lung lobes continued to exhibit an increased severity compared to the majority of lung lesions seen at 1 and 2 DPI. Bronchiolar epithelial hyperplasia and cytokaryomegaly were noted in addition to bronchiolitis.

### FDG-PET/CT and Histopathology Show Positive Correlation During Infection

**Figure 6:**
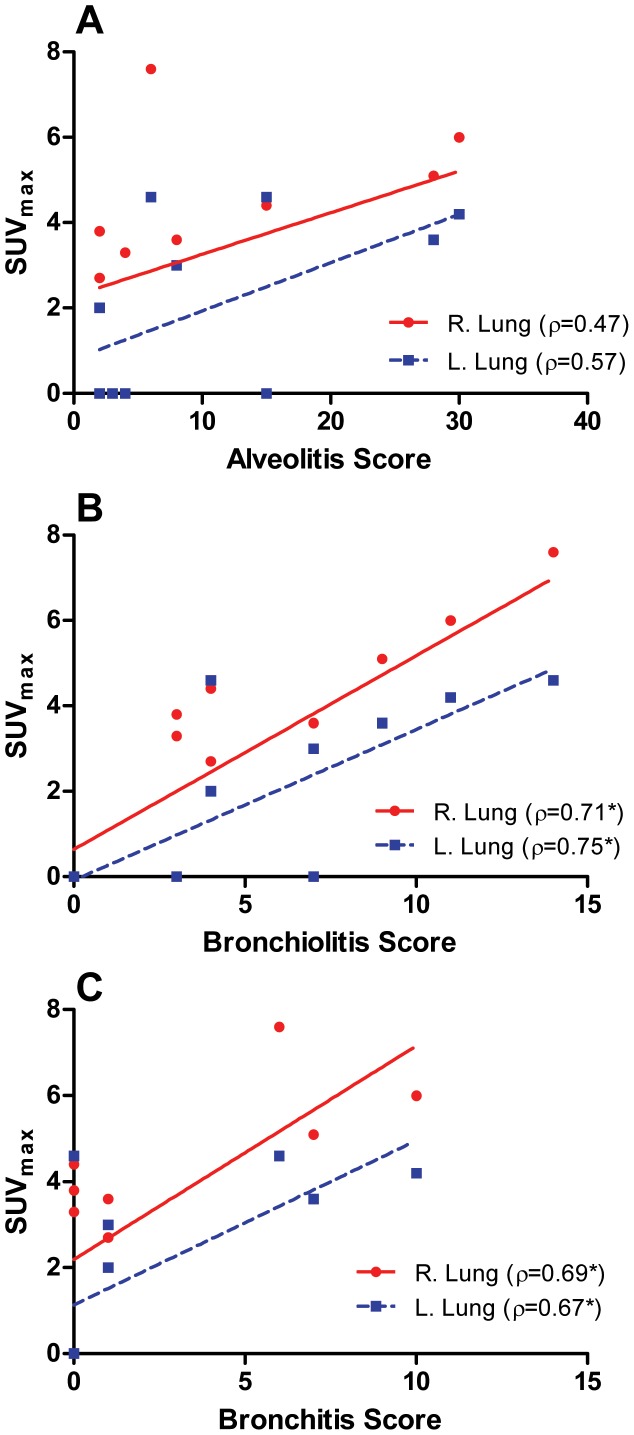
Correlations between SUVMax of lung lesions on FDG-PET versus histopathologic severity scores. A) SUVMax versus cumulative alveolitis severity shows minimal positive correlations in the right (red) and left (blue) lung. B) SUVMax versus cumulative bronchiolitis severity shows high positive correlation in both lungs. C) SUVMax versus cumulative bronchitis severity demonstrates moderate correlation.

To evaluate potential correlations between PET/CT with histopathology, the SUVMax of lesions in the right and left lung of each ferret were compared with the cumulative histopathology scores assigned by the veterinary pathologist (Fig. 6). On average, the SUVMax was higher in the right lung than the left lung but the slopes and Spearman’s correlation coefficients (*ρ)* were similar between the two sides. The highest correlation was seen between the cumulative bronchiolitis score and SUVMax (*ρ* of 0.71 and 0.75 on the right and left, respectively). The next highest was between the cumulative bronchitis score and SUVMax (*ρ* of 0.69 on the right and 0.67 on the left). A weaker positive correlation was seen between the cumulative alveolitis score and SUVMax (*ρ* of 0.47 on the right and 0.57 on the left).

## Discussion

Herein, we show for the first time the feasibility of utilizing [^18^F]-FDG PET coupled with CT imaging of H1N1pdm in ferret to track the progression of pulmonary disease in real-time. We chose a low passage clinical isolate, KY/180, which has a change in the HA1 gene, D222G. The D222G change in H1N1pdm correlates with increased severity of disease in patient cases from several countries [37]–[40]. The patient from which we obtained the KY/180 isolate also had a severe course of influenza illness over a period of 19 days that resulted in death. Recently, studies in mice and ferrets infected with pandemic influenza viruses A/California/04/2009 and A/Netherlands/602/2009 engineered with the D222G mutation have shown that the D222G mutation are lethal in mice, but not ferret [41], [42]. The lethality in mice, but not ferrets, has been attributed to the greater abundance of α2-3-SA in the mouse model [42], [43]. All of these viruses have an affinity for α2,6-SAs associated with attachment to and replication in cells of the upper respiratory tract as shown by the high levels of viral replication in the nasal turbinates. Thus, infection of ferrets with these H1N1pdm isolates engineered with D222G and our clinical isolate have not correlated with clinical findings in patients. These results in ferrets are not surprising given that 80% of the fatal cases of H1N1pdm had underlying medical conditions and bacterial infections [6]. Discovery of the molecular components of the host response that may promote pathogenesis will be critical for defining new treatments.

Noninvasive imaging can provide real-time in vivo monitoring of the progression of infection, inflammation and disease that may give insight into the mechanisms that modulate disease progression. Recently, Veldhuis Kroeze *et al*, presented data on the monitoring of pulmonary lesions of H1N1pdm influenza virus-infected ferrets with CT scanning which correlated with disease progression and severity [44]. As those studies demonstrate, CT is a powerful tool, but it will not give the molecular details that can be provided by PET or SPECT imaging of probes that target critical host responses such as neutrophil invasion. In our study we coupled CT scanning with the [^18^F]-FDG radiotracer and show infection and inflammation of influenza infection in the lower respiratory system with foci of increased [^18^F]-FDG uptake corresponding to areas of lung opacity on CT, with underlying inflammation on necropsy. In comparison to human CT imaging studies of influenza, the molecular images in the ferret show strong similarity. CT findings in patients with confirmed influenza infection show patchy ground-glass opacities in segmental multifocal distributions, mixed with areas of consolidation in the lung [12], [25], [45]. Moreover, the few case reports of human influenza in which lungs were imaged by [^18^F]-FDG PET demonstrate areas of high uptake in these ground-glass opacities and consolidation [31]. Our study similarly demonstrates this pattern in the ferret model, also showing patchy opacities on CT with high uptake of radiotracer on PET, with necroscopy-based confirmation of inflammation in the left caudal lobe. Specifically, we show the ferret lung demonstrated progressive consolidation on CT and FDG uptake on PET predominantly in the right caudal lobe, which progressed to the left caudal lobe by day 3 p.i. By day 6, the diffuse metabolically active lesions seen on PET/CT were similar to what has been reported in the human literature during the 2009 H1N1 pandemic [31].

Histopathologic evaluation of the lungs confirmed the progressive nature of the pulmonary lesions and corroborated the radiologic data. Suppurative and necrosuppurative bronchiolitis seen on days 1 and 2 became progressively worse by days 3 and 7 post-infection. The inflammation tended to be patchy or multifocal and an entire lung lobe was never uniformly affected, corresponding to the multiple patchy lesions seen on PET/CT by the end of the study. This also agreed with the analyses of the viral titers in various sections of the right caudal lobe and the PET/CT imaging. Our analyses of viral titers in the four representative sections suggest different levels of infiltration of the lobe. The histopathologic scoring for bronchiolitis correlated the best with the SUVMax of the lesions seen in the right and left lungs on PET.

In general, the severity of infection and inflammation on imaging can be represented by (1) the volume of affected lung (i.e. the percentage of diseased lung relative to total lung capacity), and (2) the extent of parenchymal destruction (disruption of pulmonary architecture) and inflammatory cell migration. Our study first aims to correlate FDG uptake measurements with histology, thereby analyzing the extent of parenchymal destruction and cellular infiltrates.

It should be considered that there can be variation in matching FDG uptake with histologic severity because more severe architectural distortion can lead to necrosis with more dead cells, therefore showing less uptake of radiotracer among metabolically inactive dead cells and nonviable tissue. Our study, however, shows that progressing inflammatory infiltrates on histology in the studied time period after acute influenza infection corresponds to radiologic trends. Additionally, our study demonstrates spatial progression with increased size and number of abnormal foci in the lung parenchyma during acute infection.

Ultimately, utilizing these new imaging tools, we envision a number of future experiments to delineate potential differences in the course of H1N1pdm and H5N1 infection in ferrets. We also plan to explore additional radiotracers that might reveal potential differences in host responses in the immune system and the process of acute injury in the lung. Future studies will assess differences in presentation of those who recover from infection versus those who eventually succumb to infection such as with more lethal isolates such as H5N1. This model should be valuable in rapid assessment of the effect of various treatments on pulmonary inflammation and damage. Finally, these first PET/CT imaging approaches could be extended to a number of other important pulmonary infections caused by pathogens such as hantaviruses, respiratory syncytial virus, and SARS CoV, to gain further insight into the spatiotemporal *in vivo* dynamics of disease progression [18].

## Materials and Methods

### Virus and Cells

The influenza H1N1pdm virus, A/Kentucky/180/2010, (KY/180; GenBank CY99332 and CY99333) was isolated from the nasal swab of a severe hospitalized case (hospitalized in March 2010) provided by the Severe Influenza Pneumonia Surveillance project, an ongoing clinical study of hospitalized patients with influenza pneumonia in Kentucky (courtesy of Dr. Julio Ramirez). The virus was isolated and passaged in the allantoic cavity of ten-day-old embryonated hens’ eggs at 37°C. The allantoic fluid was harvested 72 h after inoculation, pooled and stored in stored in aliquots at −80C until use. The infectious virus titer of the resulting seed stock was determined by TCID_50_ (50% tissue culture infectious dose) and the titer calculated by Reed and Muench [46] and confirmed by plaque assay on MDCK cells. Passage E2 was used for the studies reported herein.

### Ferrets

Ferret studies were approved by the University of Louisville Institutional Animal Care and Use Committee. University of Louisville has Veterinary Medicine tasked to monitor and support all animal experiments. Research was conducted in compliance with the Animal Welfare Act and other federal statutes and regulations relating to animals and experiments involving animals and adheres to principles stated in the *Guide for the Care and Use of Laboratory Animals*, National Research Council, 1996. The facility where this research will be conducted is fully accredited by the Association for Assessment and Accreditation of Laboratory Animal Care International.

All female Fitch ferrets were obtained from Triple F Farms (Sayre, PA). Ferrets were selected after screening blood samples for the presence of influenza antibodies using a hemagglutination inhibition assay (HI). Ferrets that were seronegative for seasonal and pandemic viruses were shipped directly to the University of Louisville Regional Biocontainment Laboratory and acclimated for seven days prior to initiation of the studies. Animals were fed Teklad Laboratory Diet #2072 (Harlan/Teklad, Madison, WI) and water *ad libitum.*

For the characterization of the progression of infection of the KY/180 clinical isolate we utilized four month old, female ferrets. Prior to infection with virus, ferrets were anesthetized with 0.05 mg/kg atropine, 5.0 mg/kg ketamine, and 0.08 mg/kg dexmedetomidine intramuscularly. Subsequently, six animals were inoculated intransally (i.n.) with 0.5 mL of infectious virus per naris as a bolus, which was diluted to 10^5.7^ TCID_50_/mL in phosphate buffered saline (PBS). Six additional animals were inoculated by i.n. with 0.5 mL of infectious virus per naris as a bolus with PBS (mock). Anesthesia was reversed with 0.4 mg/kg atipamezole. Ferrets were monitored daily for temperature and clinical symptoms. On day 2 post-infection, two ferrets were taken to measure viral titers in blood, lung, brain, trachea, nasal turbinates, spleen, kidney, thymus, liver, duodenum, jejuno-ileum, large intestine, and rectum. Gross pathology was defined during necropsy for the lung. At 14 and 28 days post-infection two additional ferrets from each group were analyzed for viral titers and pathology in the lung.

For molecular imaging studies, twelve, four-month-old female Fitch ferrets were utilized. Animals were fed food and water *ad libitum* except 4 h prior to and during CT/PET imaging. On day 0, ferrets were anesthetized prior to inoculation with virus or PBS with ketamine, dexmedetomidine and atropine. Eight animals were inoculated i.n. with 0.5 mL of infectious virus per naris as a bolus, which was diluted to 10^6.0^ TCID_50_/mL in phosphate buffered saline (PBS). Four ferrets were inoculated i.n. with 0.5 mL PBS per naris as a mock-infected control. For imaging, on days 0, 1, 2, 3 and 6, anesthetic induction and maintenance were achieved with 1–3% isoflurane. Blood glucose levels were checked prior to administration of the radiolabeled tracer to ensure that they were within normal limits, which typically range from 62–134 mg/dL in ferrets [47]. Glucose was provided to animals to compensate for body fluids lost during imaging. Typically 30 mls of Lactated Ringer’s solution (Hospira) was administered subcutaneously (s.q.) following completion of the imaging. Animals were monitored for body temperature and vital signs during imaging.

### CT and PET Imaging

Imaging was performed on 0, 1, 2, 3, and 6 days post-infection (DPI). Each day, four ferrets were imaged with CT and PET on hardware designed for preclinical animal studies, including microCT and microPET, respectively. Two ferrets were euthanized the each day and necropsied to obtain tissue samples for virologic and histopathologic analyses (please see study design Table 1). Image acquisition was conducted with a Siemens Inveon Trimodal Scanner (Siemens Preclinical, Knoxville, TN), which is a small animal imaging platform that combines microPET, microCT, and microSPECT modalities within one unit. This combination facilitated co-registration of PET and CT images as the study subject was kept in a uniform position on the scanner bed, minimizing potentially large motion artifacts as a result of repositioning the animal between each scan. The Inveon microCT scanner features a variable-focus tungsten X-ray source with an achievable resolution of 20 µm and a detector with a maximum field of view (FOV) of 8.4 cm×5.5 cm. The source-to-object distance was 263.24 mm and the source-to-detector distance was 335.67 mm. The Inveon PET detector provided an axial field of view (FOV) of 12.7 cm with a spatial resolution of 1.44 mm. PET images were reconstructed using a 2D-filtered backprojection algorithm with attenuation correction provided by microCT imaging. For the microCT scan, the following imaging settings were used: two bed positions, 80 kVp, 500 µA, 500 ms exposure time, and 4×4 binning. After each ferret underwent microCT imaging, the bed position was reset and microPET imaging with 18F-FDG (PETNET, Louisville, KY) began immediately. For each ferret, 2 mCi of 18F-FDG was administered (i.p.) with a 60–90 min uptake period. Radioactive dose was confirmed with an Atomlab 500 Dose Calibrator (Biodex Medical Systems Inc., NY).

### Image Processing and Analyses

All imaging data were processed with PMOD software (v3.1; PMOD Technologies Ltd., Zurich, Switzerland). MicroCT data were received from the Inveon platform as DICOM files and PET data as microPET files. Scans were imported into the program’s local SQL database with the units for the PET radiotracer in kBq/cc. PET images were co-registered with the CT images with re-slicing done as necessary to facilitate later calculations. For analysis of 18F-FDG levels, the standardized uptake value (SUV) was used. SUV is a widely used semi-quantitative measure that normalizes radiotracer uptake in a given region of interest based on body weight, and calculated for this study as follows:
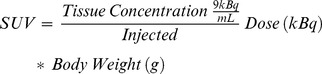
(1)


For all calculations, animal weights were expressed in kilograms and FDG activity in megabecquerels. For each image series, SUVs for each voxel were calculated using an external filter in PMOD, with the radionuclide half-life set at 6586.2 sec for 18F-FDG. For each pulmonary lesion, an ellipsoid volume of interest (VOI) was generated that encompassed the structure. Then, automatic isocontour detection was used to refit the VOI by setting a threshold of 50–60% of the difference between the maximum and minimum intensity SUVs in the ellipsoid VOI such that 0.5*(SUV_max_−SUV_min_). In cases where the automated threshold included contiguous structures in the VOI, manual refitting in conjunction with the co-registered CT scan was used to exclude those surrounding structures. For all VOIs, maximum SUV (SUV_Max_) and average and standard deviation of all pixels in the volume (SUV_Mean_ ± SD) were calculated.

For CT analysis, image interpretation was performed by a radiologist (in consultation with the scientific team) having more than ten years of diagnostic experience along with formal certifications by the American Board of Radiology (ABR) and the American Board of Nuclear Medicine (ABNM). Lesions on CT were identified using conventional criteria and terminology; Ground-glass opacity (GGO) is defined in this study as hazy increased lung opacity, with discernible underlying lung architecture such as visible bronchial and vascular structures, representing partial displacement of air in interstitial and alveolar airspaces; Consolidation is defined in this study as high density lung lesions (more dense than GGO) in which vascular and bronchial margins are obscured, representing complete displacement of alveolar air [27].

### Viral Titers in Swabs and Tissues

On days 1, 2, 3 and 6 and prior to euthanasia, swabs were taken from each ferret from nasal, throat and rectal regions. Following scheduled euthanasia, the nasal turbinates and the right caudal lobe of the lung from each ferret, which was divided laterally into four segments, were isolated. All swab and tissue samples were snap-frozen in liquid nitrogen and stored at −80°C until analyzed for virus titer by TCID_50_. Frozen tissues were weighed and diluted 10% weight per volume into cold DMEM with 1% penicillin/streptomycin and 0.2% BSA before being homogenized and centrifuged to remove debris. Tissue homogenate and swabs were serially diluted 10-fold in DMEM with 2 µg/mL TPCK-Trypsin, 0.2% BSA, 4.5 g/L glucose, 1% penicillin/streptomycin, 2 mM L-glutamine, and 25 mM HEPES. Each sample was analyzed in quadruplicate following incubation in 96-well plates with Madin-Darby canine kidney (MDCK) cell monolayers at 37°C in 5% CO_2_ for three days. Supernates were collected from each well were assayed for hemagglutination activity using 0.5% turkey red blood cells as an indicator of infection. Viral titers were expressed as log_10_ TCID_50_/mL and were calculated using the Reed-Muench method [48].

### Hemagglutination Inhibition Assay (HI)

The HI test quantitates serum antibody to influenza virus which can prevent agglutination of turkey RBCs (Fitzgerald Industries International Inc., MA). Heat-inactivated serum samples were treated with receptor-destroying enzyme (Sigma-Aldrich) for removing nonspecific inhibitors (followed by RBC adsorption) and were diluted 2- fold serially from initial dilution of 1∶10. HA antigen (8 HA units in 25 µL) were added onto each well and incubated for 1 h at RT. Following antigen-antibody reaction, 50 µL of 0.5% turkey RBC were added to each well and incubated for 1 h at RT. HI negative wells were scored based upon a diffuse sheet of agglutinated RBCs covering the bottom. HI positive wells were scored if they showed a well circumscribed button of nonagglutinated RBCs.

### Histopathology

Lungs were inflated and stored in 10% neutral-buffered formalin. Three lung sections were placed into cassettes per lung section (right cranial, left cranial, left caudal, and right middle lobe) until they were trimmed, paraffin-embedded, and sectioned. Sections were mounted on glass slides and stained with hematoxylin and eosin for microscopic evaluation at Experimental Pathology Laboratories, Inc. by a veterinary pathologist. Sections were examined for the presence of abnormal findings including supprative and necrosupprative inflammation; epithelial hyperplasia and cytokaryomegaly; and fibrinous and exudative changes. Changes were graded with a standardized scale of 0–5, with 0 classified as “not present”, 1 as “minimal”, 2 as “slight/mild”, 3 as “moderate”, 4 as “moderately severe”, and 5 as “severe/high.” For each ferret, a composite score for pathological changes was generated based on the locations in the respiratory tract (alveoli, bronchioli, bronchi) for statistical evaluation.

### Statistical Analyses

All statistics were performed using R version 2.13.0 and GraphPad Prism 5. For each image, mean SUVMax and standard deviations were obtained. For each ferret, SUVMax values were correlated with histopathologic scoring using Spearman’s Rho (*ρ*).
